# Traditional medicine use among rabies exposed individuals in Ethiopia: A systematic review and meta-analysis

**DOI:** 10.1371/journal.pntd.0013319

**Published:** 2025-07-11

**Authors:** Gizew Dessie Asres, Yeshiwork Kebede Gessesse, Desalew Salew, Negese Sewagegn Semie, Etsegenet Kindie, Molalign Tarekegn Minwagaw, Wudu Tafere, Zewudu Belay, Habtamu Alebachew, Getasew Mulat Bantie, Taye Zeru

**Affiliations:** 1 Health Research Development Directorate, Amhara Public Health Institute, Bahir Dar, Amhara, Ethiopia; 2 Integrated Oby-Gyn Emergency Surgeon, Durbete primary Hospital, Durbete, Amhara, Ethiopia; 3 Regional Data Management Center Directorate Director, Amhara Public Health Institute, Bahir Dar, Amhara, Ethiopia; 4 Human Rabies Laboratory Focal, Amhara Public Health Institute, Bahir Dar, Amhara, Ethiopia; 5 Animal Rabies Focal, Amhara Livestock and Fishery Resource Development Office, Bahir Dar, Amhara, Ethiopia; 6 One Health Focal, Amhara Public Health Institute, Bahir Dar, Amhara, Ethiopia; 7 Regional Data Management Center Directorate, Amhara Public Health Institute, Bahir Dar, Amhara, Ethiopia; Mizan-Tepi University, ETHIOPIA

## Abstract

**Background:**

Worldwide, traditional medicine (TM) is an important and often underestimated part of health services. TM, of proven quality, safety, and efficacy, contributes to the goal of universal health coverage. However, no TM is approved worldwide for rabies treatment. Rabies is almost 100% fatal once symptomatic, leading to acute encephalitis. The challenges of rabies prevention and control in Ethiopia are multifaceted. Successful prevention and control of rabies requires the collaboration of important stakeholders with the One Health approach. But in Ethiopia, the one health model has missed a very important player in rabies control intervention, the traditional healers. This review aims to assess the proportion of individuals visiting traditional healers to provide evidence-based recommendation.

**Method:**

We prepared a review protocol per Joanna Briggs Institute (JBI) manual for evidence synthesis and conducted a comprehensive search of PubMed, Cochrane, Google Scholar and African Index Medicus databases and grey literature from 17 December 2023–30 January 2024. The pooled proportion of traditional medicine use among rabies-exposed individuals was computed using R v 4.3.1 software. Subgroup analysis was done on sample size, geographical location and year of publication. Publication bias was assessed using a funnel plot.

**Result:**

The pooled proportion of traditional medicine use following rabies exposure was 0.57 at 95% CI (0.45-0.69) for the random effects model with I^2^ = 98% and p < 0.01. To investigate the source of heterogeneity, sub-group analysis has been done on sample size, study area, and year of publication. All the above-listed variables were significant sources of statistical heterogeneity. Of which year of publication from 2019-2023 (I^2^ = 99%, p < 0.01), Studies from Oromia regional state (I^2^ = 99%, p < 0.01) and study sample size >500 (I^2^ = 99%, p < 0.01) were the highest source of heterogeneity.

**Conclusion:**

More than half of rabies-exposed individuals visit traditional healers. This implies that significant healthcare demand related to rabies is addressed by traditional healers, even though the quality is not yet audited. The EMOH in collaboration with partners should work on the integration of traditional healers for rabies prevention and control interventions as One Health stakeholder.

## Author summary

Traditional medicine plays important role in healthcare across many countries, including Ethiopia, though it is often ignored by the formal health system. In Ethiopia, traditional remedies are commonly used for a range of illnesses, including rabies exposure, despite no scientific evidence supporting traditional treatments for rabies, a disease that is almost always fatal once symptoms begin. Ethiopia uses the “One Health” strategy to prevent and control rabies through collaboration across sectors. However, traditional healers have not been included in this approach as they have greater contribution for delaying the access of post exposure prophylaxis. This review aimed to determine how many people exposed to rabies seek help from traditional healers to facilitate evidence informed decision making. Authors conducted review of both published and unpublished studies in Ethiopia their analysis showed that 57% of individuals exposed to rabies visited traditional healers, either before or instead of seeking modern medical care. This implies that traditional healers play a major role in how communities respond to rabies. Therefore, we recommend that the Ministry of Health involve traditional healers in rabies prevention and control as part of the One Health strategy to improve awareness and access to timely post exposure prophylaxis service.

## Introduction

Traditional medicine (TM) has a long history and is defined as the total of the knowledge, skill, and practices based on the theories, beliefs, and experiences indigenous to different cultures, whether explicable or not, used in the maintenance of health as well as in the prevention, diagnosis, improvement or treatment of physical and mental illness [1].

Traditional medicine (TM) is either the primary mode of healthcare delivery or serves as a complementary component in many countries around the world. It constitutes a significant, yet often underestimated, part of health services. When demonstrated to be of proven quality, safety, and efficacy, TM can contribute meaningfully to the goal of universal health coverage. Globally, all 194 WHO Member States have reported the use of TM within their communities. A recent review found that TM usage ranged from 24% to 71.3% across 14 different countries. Some studies have also shown that communities in various parts of the world utilize TM in response to rabies exposure. However, no TM intervention has been globally approved for post-exposure treatment of rabies. Countries such as Vietnam have begun integrating traditional healers into the One Health framework to support rabies prevention and control, demonstrating promising progress in this area [2–4].

In Ethiopia, TM is trusted and widely used by the community and serves as a primary source of healthcare. A recent review reported that more than 65% of the population relies on TM for addressing various health problems. Specifically, scholars have identified over twelve traditional medicinal plants used in the treatment of rabies. However, the quality, safety, and efficacy of these anti-rabies remedies have not yet been systematically evaluated or validated through scientific auditing [5].

Rabies, a zoonotic disease, is caused by a non-segmented, negative sense, single-stranded RNA virus of the family Rhabdoviridae. The incubation period ranges from a few days to a year or more, typically falling between 20 and 90 days [6]. Despite being a preventable disease, rabies is almost 100% fatal once symptomatic, leading to acute encephalitis. It affects almost all mammals, with transmission to humans primarily occurring through contact with rabid mammals such as dogs, cats, and foxes, with dogs serving as the primary vectors worldwide (99%) [7]. The rabies virus is transmitted through direct contact with saliva or brain/nervous system tissue from an infected animal, usually via a bite. Although rare, non-bite exposures, such as scratches or abrasions, can also transmit the virus [8].

Rabies is present on all continents, except Antarctica and is currently responsible for more than 60,000 human deaths per year, with children constituting over 40% of the fatalities. The majority of cases occur in poor rural communities in Africa and Asia, far from medical and veterinary services, resulting in an estimated 3.7 million disability-adjusted life years (DALYs) and economic losses of 8.6 billion USD annually worldwide [9,10]. In Asia and Africa, dog-mediated rabies imposes a major burden, with estimated human deaths of 35,172 and 21,476 annually, respectively [11].

Successful prevention and control of rabies require the involvement of relevant stakeholders from human, animal, and environmental health sectors. In 2015, the world set a goal of zero human deaths from dog-mediated rabies by 2030. To achieve this goal, four organizations— the World Health Organization (WHO), the World Organization for Animal Health (OIE), the Food and Agriculture Organization of the United Nations (FAO), and the Global Alliance for Rabies Control (GARC)—formed the United Against Rabies collaboration [12,13].

In Africa, where approximately 21,476 human deaths occur annually due to dog-mediated rabies, intensive awareness campaigns, achieving 70% dog vaccination coverage, and improving access to post-exposure prophylaxis (PEP) could significantly reduce fatalities. However, rabies remains a significant public health concern in Ethiopia, one of the countries with the highest rabies death rates globally. With over 2,700 human deaths each year, approximately eight deaths daily, Ethiopia suffers an estimated annual loss of US$114,881,471 due to rabies. Limited reporting and diagnostic facilities contribute to the underestimation of rabies cases, while inadequate availability of PEP exacerbates the situation. Despite over 88% of rabies cases being dog-mediated, dog vaccination coverage is below 20% in urban areas and virtually non-existent in rural areas [11,14,15].

Historically, rabies in Ethiopia has been a persistent health threat, primarily transmitted through dog bites. The disease was known in ancient Ethiopian medical texts, though its scientific understanding was limited. In the early 20th century, the lack of effective vaccines and the absence of formal control programs led to widespread rabies in both animals and humans, particularly in rural areas. During the 1960s and 1970s, Ethiopia began to recognize rabies as a public health issue, but resources for control were limited. In the 1980s, international organizations supported mass dog vaccination campaigns, but challenges in infrastructure and accessibility persisted. Rabies remains endemic, though control efforts have improved in recent decades [16].

Currently, rabies prevention in Ethiopia focuses on a combination of mass dog vaccination campaigns, public education, and post-exposure prophylaxis (PEP). The government, in collaboration with international organizations like the World Health Organization (WHO) and the Pan-African Rabies Control Network (PARACON), has launched initiatives to vaccinate dogs in high-risk areas, especially in rural regions with high stray dog populations. Public awareness campaigns aim to educate communities about rabies transmission, the importance of vaccination, and seeking timely medical treatment after animal bites. Additionally, health facilities offer PEP, including rabies vaccines and rabies immunoglobulin, though access remains limited in remote areas. Despite progress, gaps in healthcare infrastructure and the high number of unvaccinated dogs remain significant barriers to complete rabies control [17].

The challenges of rabies prevention and control in Ethiopia are multifaceted. A lack of comprehensive national epidemiological data, coupled with limited awareness among the population, impedes control efforts. Additionally, the absence of a well-organized surveillance and notification system hinders effective tracking and response to rabies cases [18,19]. Utilizing the One Health model, which integrates human, animal, and environmental health, is essential for effective rabies elimination, focusing on prevention (e.g., dog bite prevention and mass dog vaccination) and response (e.g., bite management and post-exposure prophylaxis) [20].

Recognizing the importance of multisectoral collaboration, the Government of Ethiopia, and its development partners have designed a five-year (2018–2022) One Health national strategic plan and a national rabies control and elimination strategic plan (2018–2030) [21,22]. But the important stockholders in rabies prevention and control, the traditional healers, have been missed.

Managing individuals exposed to rabies involves prompt post exposure prophylaxis vaccine to prevent disease onset. One objective of the strategic plan to end human deaths from dog-mediated rabies is to increase access to PEP and wound care for those exposed, alongside mass dog vaccination [12]. But pocket studies in different parts of Ethiopia indicated that the community is using TM for post rabies exposure to prevent onset of the disease even though the quality, safety, and efficacy is not yet scientifically evaluated [23]. This review aims to assess the pooled proportion of individuals who seek care from traditional healers and to provide evidence-based recommendations for inclusion of traditional healers as key stakeholders in national rabies elimination strategies.

### Review question

This systematic review was guided by a review question ‘What is the pooled proportion of traditional medicine use among rabies exposed individuals in Ethiopia?’

## Methods

### Study area

Ethiopia, located in the Horn of Africa, is the second-most populous country in Africa, with over 120 million people as of 2023. It is characterized by diverse geographical features, ranging from highland plateaus to lowland deserts, and is home to a wide variety of ethnic groups, languages, and cultural practices. Politically, the country has a federal government system and is divided into regional states based on ethnicity. There are over 80 recognized ethnic groups and as many languages, making it one of the most linguistically diverse countries in the world [24–26]

### Study design

A systematic review and meta-analyses using condition, context and population (COCOPOp) framework of Joanna Briggs Institute (JBI) and based on PRISMA guideline was done to assess the proportion of traditional medicine use among rabies exposed individuals in Ethiopia. A review protocol was developed using JBI manual for evidence synthesis to pre-define the objectives and methods of the systematic review. The protocol was registered from International Prospective Register of Systematic Reviews (PROSPERO) as ID = CRD42022313036.

### Search strategy and selection process

We conducted a thorough examination of the PROSPERO database (http://www.library.ucsf.edu/) to ascertain the presence of any published or ongoing projects related to our chosen topic, with the aim of preventing any unintended duplication of efforts. Our investigation yielded no registered reviews, whether in progress or completed, within the domain of our selected subject matter. Then, we conducted a comprehensive search of PubMed, Cochrane, Google Scholar and African Index Medicus databases and grey literatures from 17 December 2023–30 January 2024. The following was searching strings used for PubMed search and was modified accordingly for other data bases: rabies OR “rabies Virus” OR “rabies disease” AND exposed OR Victims OR suspected AND patients OR individuals OR clients AND “traditional medicine” OR “traditional treatment” OR “traditional therapy” OR “traditional remedy” OR “alternative medicine” OR “complementary medicine” OR “herbal medicine” AND use OR practice OR visit OR preference AND Ethiopia (S1 Annex).

### Eligibility criteria

The inclusion criteria were based on COCOPOp framework and the outcome variable; proportion of traditional medicine use among rabies exposed individuals (Table 1).

**Table 1:** Inclusion and exclusion criteria of articles, 2024.

Included studies	Excluded studies
Rabies exposure studies that assessed traditional medicine use in Ethiopia using any design and setting. Rabies exposure studies that assessed health seeking behavior in Ethiopia using any design and setting. Studies written in English language.	Rabies exposure studies that do not assess where victims got care in Ethiopia. Experimental studies on rabies virus pathogenesis, vaccine trial and diagnosis. Rabies exposure studies that have no free full text access.

### Study Screening and Selection

The process of screening was conducted in two levels. Level one was based on titles and abstracts of the articles. In the second level, articles that have passed the screening level were downloaded and evaluated against the eligibility criteria. All selected articles from this stage were saved in a separate folder for quality assessment and were used for evidence synthesis. Those articles excluded based on the full-text assessment against the inclusion and exclusion criteria were justified. The entire screening and selection process was undertaken using Rayyan software [27] independently by two authors, who later compared their results from the software and there were no major discrepancies and minor discrepancies were resolved based on consensus between the two authors.

### Quality assessment of studies

Critical appraisal tool based on the study design was used to assess the methodological quality of studies. A critical appraisal checklist developed by JBI for prevalence studies have been used for this study (S2 Annex). Two reviewers GDA and YKG appraised the studies independently using the tool. The tool encompassed 9 criteria for rating different quality elements. During quality assessment, disagreements were resolved using weighted kappa index, which was 98.33% and the two reviewers reached an agreement [28].

### Data extraction

The JBI Standardized data extraction template was used to ensure the extraction of the same types of data across the included studies. The two reviewers GDA and YKG extracted the entire necessary data independently using systematic review data repository plus (SRDR+) online platform. Data was collected on; study details, study method, and results on proportion of traditional medicine use.

### Descriptive synthesis and meta-analysis

The data synthesized for this systematic review were the results extracted from individual research studies relevant to the review question. Narrative summary in this review utilized tables, graphs, and other diagrams in addition to textual descriptives to understand how studies compare to each other. It included the presentation of the quantitative results reported in individual studies; the point estimates and the interval estimate at 95% confidence intervals for traditional medicine use among rabies exposed individuals. Pooling of the quantitative results were done under R version 4.3.1 software [29] using fixed and random effects model. The extent of heterogeneity across studies was checked using Q-test and I²-test (I squared >50% indicating significant heterogeneity). Subgroup analysis was conducted on sample size of individual studies, year of publication and location.

## Results

### Search process and results

**Fig 1:**
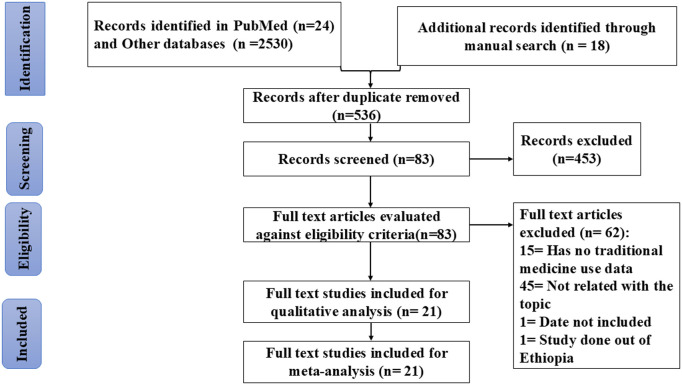
Flowchart presenting the study selection with the Preferred Reporting Items for systematic Review and Meta analysis (PRISMA).

During the bibliographic data base search, a total of 2, 572 studies were identified. Of which 2, 036 were duplicates and removed. Of 536 remaining studies, 453 were found to be unrelated the review question and excluded based on title and abstract screening. Finally,83 full text articles were downloaded for further evaluation against eligibility criteria and 15 full text articles excluded as they missed data on proportion of traditional medicine use and 45 full text articles found unrelated for this systematic review and excluded. One full text article was excluded due to date not included and one article was excluded due to study location is outside Ethiopia. At last, we ended up with 21 full texts articles for evidence synthesis (Fig 1).

### Characteristics of included studies

Among 21 included studies published from 2013-2023 in Ethiopia, the smallest sample size was 107 on a study done in Oromia and the largest one was 1440 on a study done in Tigray regional state. The study settings included were at community level except single study done at health facility in Oromia. These studies involved a total of 9261 participants (Table 2).

**Table 2:** Key characteristics of included studies for proportion of traditional medicine use among rabies exposed individuals in Ethiopia, 2024.

Study_ID	Pub. year	Study Location	Study Setting	Study Design	Sample Size	Got care from	
Health Facility	Traditional Healer
Tadesse_2014 [30]	2014	Amhara	community	Cs	410	162	248
Ebuy_2019 [31]	2019	Tigray	Community	Cs	1440	573	867
Awoke_2015 [32]	2016	Amhara	Community	Cs	384	144	240
Nigatu_2016 [33]	2016	Amhara	Community	Cs	416	175	228
Nejash_2017 [34]	2017	Oromia	Community	Cs	135	89	41
Tariku_2017 [35]	2017	Oromia	Community	Cs	384	145	176
Addis_2019 [36]	2019	Amhara	Community	Cs	384	343	26
Yigardush_2017 [37]	2017	Amhara	Community	Cs	360	193	167
Eyob_2016 [38]	2016	Addis_Ababa	Community	Cs	384	266	77
Adane_2022 [39]	2022	Amhara	Community	Cs	899	555	344
Amare_2020 [40]	2020	Amhara	Community	Cs	384	117	267
Haben_2020 [41]	2020	Tigray	Community	Cs	399	173	226
Shumye_2016 [42]	2016	Amhara	Community	Cs	139	75	64
Tadele_2015 [43]	2015	Oromia	Health Facility	Cs	384	93	291
Hunde_2023 [44]	2023	Oromia	Community	Cs	384	86	298
Tamiru_2017 [45]	2017	Oromia	Community	Cs	406	38	368
Reta_2015 [46]	2015	Amhara	Community	Cs	400	155	245
Tsegaye_2016 [47]	2016	Oromia	Community	Cs	803	32	743
Balako_2019 [48]	2019	Oromia	Community	Cs	107	64	43
Wudu_2013 [49]	2013	Amhara	Community	Cs	120	19	101
Rea_2016 [50]	2016	Oromia	Community	Cs	539	217	322

### Methodological quality of included studies

Methodological quality assessment was done using the JBI’s Prevalence Studies Critical Appraisal Checklist. Assessment was done by two independent assessors, GDA and YKG. Discrepancies between assessors were resolved through consensus and mutual understanding. The assessment results revealed that quality of included studies range from 66.7% to 100% (S3 Annex).

### Findings of the review

**Fig 2:**
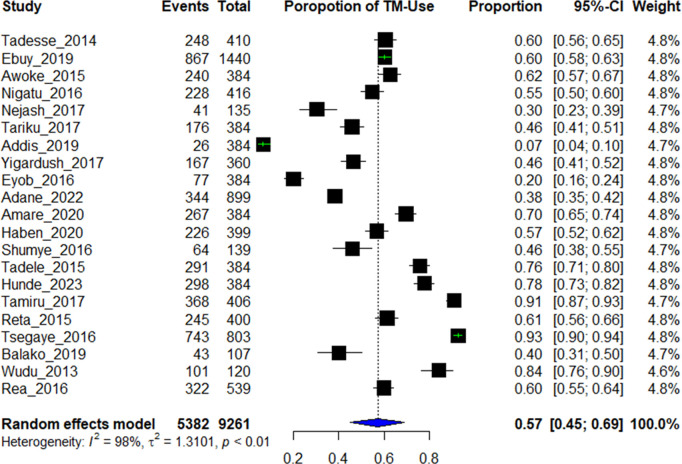
Forest plot of included studies for proportion of traditional medicine use among rabies exposed individuals in Ethiopia, 2024.

From 21 included studies the pooled prevalence of traditional medicine use following rabies exposure was 0.57 at 95% CI (0.45-0.69) for the random effects model with I^2^ = 98% and p < 0.01 as shown in Fig 2 below.

### Publication bias analysis of included studies

**Fig 3:**
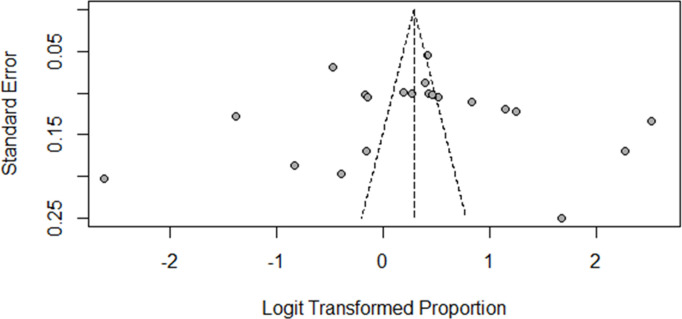
Funnel plot of included studies for proportion of traditional medicine use among rabies exposed individuals in Ethiopia, 2024.

The funnel plot indicates no asymmetry in the distribution of effect sizes, suggesting the absence of publication bias among the included studies (Fig 3).

### Sub group analysis

**Fig 4:**
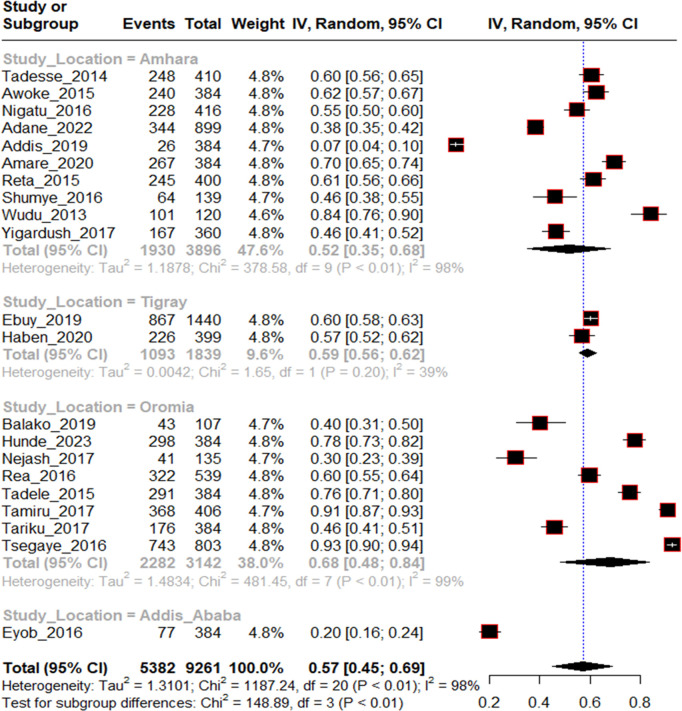
Sub-group analysis of included studies by location for proportion of traditional medicine use among rabies exposed individuals in Ethiopia, 2024.

**Fig 5:**
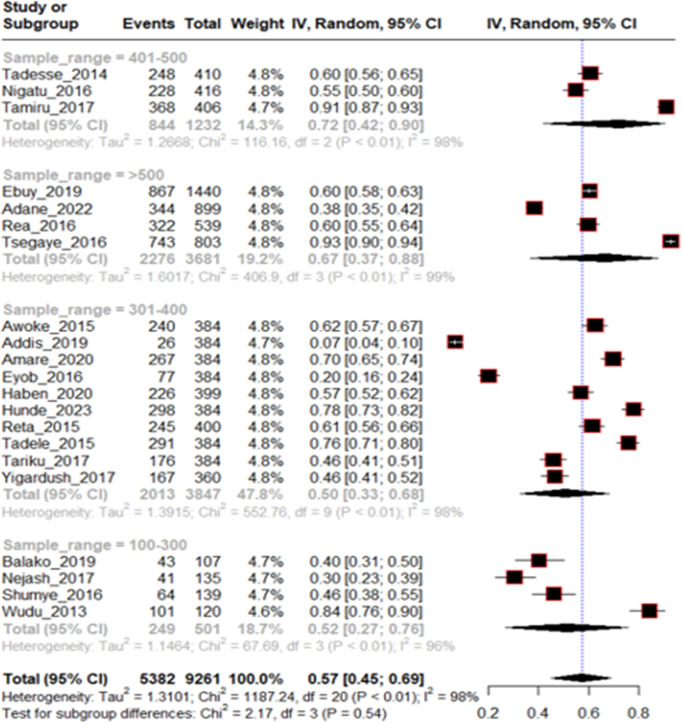
Subgroup analysis by sample size of included studies for proportion of traditional medicine use among rabies exposed individuals in Ethiopia, 2024.

**Fig 6:**
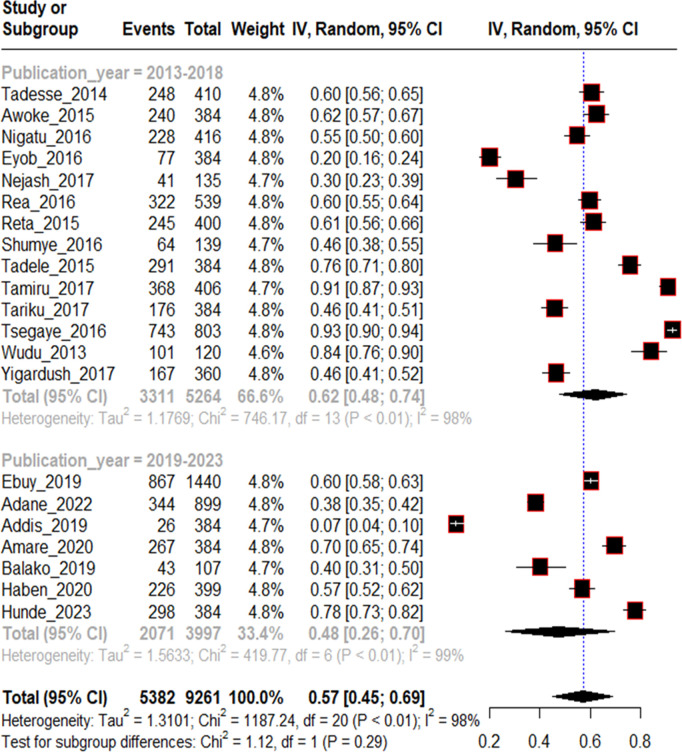
Subgroup analysis of included studies by publication year for proportion of traditional medicine use among rabies exposed individuals in Ethiopia, 2024.

To investigate source of heterogeneity, sub group analysis was done on sample size, study area, and year of publication. All these variables were not significant source of statistical heterogeneity. Of which year of publication from 2019-2023 (I^2^ = 99%, p < 0.01), Studies from Oromia regional state (I^2^ = 99%, p < 0.01) and study sample size >500 (I^2^ = 99%, p < 0.01) were highly heterogeneous within themselves (Figs 4–6).

## Discussion

The distribution of 5382 traditional medicine users from 21 included studies throughout Ethiopia was 35.9%, 42.4%, 20.3% and 1.4% in Amhara, Oromia, Tigray and Addis Ababa respectively. The smallest sample size among included studies was 107 and the largest one was 568 from studies done in Addis Ababa and Amhara respectively. Majority, 66.6%, of included articles were publications of 2013–2018 period. The highest proportion of traditional medicine use following rabies exposure was 93% and the lowest was 7% as reported by studies done in Oromia in 2016 and Amhara in 2019 respectively.

The aim of this systematic review was to assess the pooled proportion of traditional medicine use among rabies exposed individuals in Ethiopia. Accordingly, the pooled proportion was 0. 57 (95% CI: 0.45 – 0.69) for random effect model at I- squared (inconsistency) = 98%, P < 0.01). This implies that traditional healers served more rabies exposed individuals than health facilities did, even though the service quality provided by traditional healers is not yet scientifically audited with controlled trials.

This finding, 57% traditional medicine use following rabies exposure, was higher than a study done in Pakistan (38.7%) [51], Nepal (5.66 to 8.2%) [52,53], Mogadishu (3.6%) [54], Nigeria (18.5% to 27%) [55,56] and Mozambique (4.7%) [57] but lower than study done in Colombia (87.4%) [58]. On the other hand, this study is lower than general TM use proportion in Ethiopia (65%) [5] and it is within the range of global TM use proportion (24-71.3%) [3] based on 14 countries. The difference may be due to variations in characteristics of study participants, socio economic status of countries, access to health facility, health literacy of the community and variations in study designs and types (systematic review and single original study).

The study’s results also highlight important health implications. The use of traditional medicine after rabies exposure could have serious consequences for public health. If individuals are relying on traditional remedies instead of seeking appropriate medical care, it may delay or prevent them from receiving the necessary treatment, increasing the risk of fatal outcomes.

The finding revealed that the proportion of traditional medicine use was significantly different across regions. The proportion of traditional medicine use following rabies exposure was 0.59 (95% CI:0.56-0.62), 0.52 (95% CI:0.35-0.68), 0.68 (95% CI:0.48-0.84) and 0.20 (95% CI:0.16-0.24) in Tigray, Amhara, Oromia and Addis Ababa respectively. This variation may be due to cultural differences across regions. The proportion of traditional medicine use post rabies exposure showed slight decrement from 2013 to 2023. It was 0.62 (95% CI:48–74) with I^2^ = 98% and 0.48 (95%CI:0.26-0.70) with I^2^ = 99% from 2013-2018 and 2019–2023 categories respectively. This may be due to health education and awareness changes over time.

## Limitation

A limitation of this review was the heterogeneity of the included studies in terms of study procedures, participant characteristics, and study settings. As a result, the findings were summarized at a broader level, which may obscure some of the unique features of the different approaches. Additionally, the review focused solely on the prevalence of traditional medicine use, without addressing its underlying determinants. This review also relied solely on articles published in English, and the high heterogeneity among the included studies may undermine the reliability of the pooled estimate and limit the validity of the conclusions.

## Conclusion

The finding that 57% of rabies-exposed individuals in Ethiopia seek traditional healing rather than modern healthcare highlights a significant gap in the country’s rabies prevention and treatment efforts. Despite the availability of effective vaccines and post-exposure prophylaxis, a substantial portion of the population continues to rely on traditional remedies, likely due to factors such as cultural beliefs, limited healthcare access, and a lack of awareness about the effectiveness of modern treatments. This underscores the need for targeted public health interventions that integrate traditional healing practices with modern medical care, improve awareness about rabies prevention, and enhance access to healthcare, especially in rural areas. Addressing these issues is crucial to reducing the burden of rabies in Ethiopia and ensuring timely, life-saving interventions for exposed individuals.

## Recommendation

Authors recommendations to health authorities aim to improve rabies prevention and treatment through a balanced approach that acknowledges the role of traditional medicine while promoting modern healthcare solutions based on the findings of this study:

Promote Awareness and Education: Launch culturally-sensitive public health campaigns to educate communities about the importance of seeking timely modern medical care, especially rabies vaccination and post-exposure prophylaxis (PEP).

Integrate Traditional and Modern Medicine: Train traditional healers to recognize rabies symptoms and refer individuals for timely medical treatment, fostering collaboration between traditional and modern healthcare systems.

Improve Healthcare Access: Expand access to rabies vaccines and medical treatment in rural areas to reduce reliance on traditional remedies.

Encourage Further Research: Investigate the reasons behind the preference for traditional medicine to inform more targeted health interventions and policies.

Strengthen Policy Support: Allocate resources for rabies control, including mass vaccination and healthcare infrastructure, in collaboration with international and local organizations.

## Supporting information

S1 Annex PubMed search strategy. (DOCX)

S2 Annex Quality appraisal checklist. (DOCX)

S3 Annex Methodological quality of included studies for proportion of traditional medicine use among rabies exposed individuals in Ethiopia, 2024. (DOCX)

S1 File PRISMA_2020_checklist-TM use. (DOCX)

S2 File Data extracted from included studies. (XLSX)
