# Supplementary material for: Traditional medicine use among rabies exposed individuals in Ethiopia: A systematic review and meta-analysis
Source: PLoS Negl Trop Dis. 2025 Jul 11;19(7):e0013319. doi: 10.1371/journal.pntd.0013319 (PMC12273941; doi:10.1371/journal.pntd.0013319)
Supplement: S1 Annex — (DOCX) [file pntd.0013319.s001.docx]

**Annex 1**: **PubMed search strategy**

Date: December 17, 2023

| Search | Search Query | Result | Time |
| --- | --- | --- | --- |
| #1 | **Search: (((((rabies) OR (rabies virus)) OR (rabies disease)) AND (exposed)) OR (Victims)) OR (suspected)** | [333,873](https://pubmed.ncbi.nlm.nih.gov/?term=%28%28%28%28%28rabies%29+OR+%28rabies+virus%29%29+OR+%28rabies+disease%29%29+AND+%28exposed%29%29+OR+%28Victims%29%29+OR+%28suspected%29&ac=no&sort=relevance) | 04:50:58 |
| #2 | **Search: (((((((((patients) OR (individuals)) OR (clients)) AND (traditional medicine)) OR (traditional treatment)) OR (traditional therapy)) OR (traditional remedy)) OR (alternative medicine)) OR (complementary medicine)) OR (herbal medicine)** | [896,808](https://pubmed.ncbi.nlm.nih.gov/?term=%28%28%28%28%28%28%28%28%28patients%29+OR+%28individuals%29%29+OR+%28clients%29%29+AND+%28traditional+medicine%29%29+OR+%28traditional+treatment%29%29+OR+%28traditional+therapy%29%29+OR+%28traditional+remedy%29%29+OR+%28alternative+medicine%29%29+OR+%28complementary+medicine%29%29+OR+%28herbal+medicine%29&ac=no&sort=relevance) | 04:55:07 |
| #3 | **Search: (((((((use) OR (practice)) OR (visit)) OR (preference)) AND (determinants)) OR (factors)) OR (predictors)) AND (Ethiopia)** | [23,806](https://pubmed.ncbi.nlm.nih.gov/?term=%28%28%28%28%28%28%28use%29+OR+%28practice%29%29+OR+%28visit%29%29+OR+%28preference%29%29+AND+%28determinants%29%29+OR+%28factors%29%29+OR+%28predictors%29%29+AND+%28Ethiopia%29&ac=no&sort=relevance) | 04:58:53 |
| #4 | **Search: ((#1) AND (#2)) AND (#3)** | [24](https://pubmed.ncbi.nlm.nih.gov/?term=%28%28%231%29+AND+%28%232%29%29+AND+%28%233%29&ac=no&sort=relevance) | 05:08:19 |
