# Supplementary material for: Traditional medicine use among rabies exposed individuals in Ethiopia: A systematic review and meta-analysis
Source: PLoS Negl Trop Dis. 2025 Jul 11;19(7):e0013319. doi: 10.1371/journal.pntd.0013319 (PMC12273941; doi:10.1371/journal.pntd.0013319)
Supplement: S3 Annex — (DOCX) [file pntd.0013319.s003.docx]

**Annex 3: Methodological quality of included studies, 2023**

| Study ID | Was the sample frame appropriate to address the target population?(1) | Were study participants sampled in an appropriate way?(2) | Was the sample size adequate?(3) | Were the study subjects and the setting described in detail?(4) | Was the data analysis conducted with sufficient coverage of the identified sample?(5) | Were valid methods used for the identification of the condition?(6) | Was the condition measured in a standard, reliable way for all participants?(7) | Was there appropriate statistical analysis?(8) | Was the response rate adequate, and if not, was the low response rate managed appropriately?(9) | Total Yes/comment |
| --- | --- | --- | --- | --- | --- | --- | --- | --- | --- | --- |
| *Tadesse_2014* | *Yes* | *Yes* | *Yes* | *Yes* | *Yes* | *Yes* | *No* | *Yes* | *Yes* | *88.9%* |
| *Ebuy_2019* | Yes | Yes | Yes | Yes | Yes | Yes | Yes | Yes | Yes | *100%* |
| Awoke_2015 | No | Yes | Yes | Yes | Yes | Yes | Yes | Yes | Yes | 88.9% |
| Nigatu_2016 | Yes | Yes | Yes | Yes | Yes | Yes | No | Yes | Yes | 88.9% |
| Nejash_2017 | No | Yes | Yes | No | Yes | Yes | Yes | Yes | Yes | 77.8% |
| Tariku_2017 | No | Yes | Yes | Yes | Yes | Yes | Yes | Yes | Yes | 88.9% |
| Addis_2019 | Yes | Yes | Yes | Yes | No | Yes | Yes | Yes | Yes | 88.9% |
| Yigardush_2017 | No | Yes | No | Yes | Yes | Yes | Yes | Yes | Yes | 77.8% |
| Eyob_2016 | No | Yes | No | Yes | No | Yes | Yes | Yes | Yes | 66.7% |
| Adane_2022 | Yes | Yes | Yes | Yes | Yes | Yes | Yes | Yes | Yes | 100% |
| Amare_2020 | Yes | Yes | Yes | Yes | Yes | Yes | Yes | Yes | Yes | 100% |
| Haben_2020 | Yes | Yes | Yes | Yes | No | Yes | Yes | Yes | Yes | 88.9% |
| Shumye_2016 | No | Yes | Yes | Yes | No | Yes | Yes | Yes | Yes | 77.8% |
| Tadele_2015 | Yes | Yes | Yes | Yes | Yes | Yes | Yes | Yes | Yes | 100% |
| Hunde_2023 | No | Yes | Yes | No | Yes | No | Yes | Yes | Yes | 66.7% |
| Tamiru_2017 | Yes | Yes | Yes | Yes | Yes | Yes | Yes | Yes | Yes | 100% |
| Reta_2015 | Yes | Yes | Yes | Yes | Yes | Yes | Yes | Yes | Yes | 100% |
| Tsegaye_2016 | Yes | Yes | Yes | Yes | Yes | Yes | Yes | Yes | Yes | 100% |
| Balako_2019 | No | Yes | No | Yes | No | Yes | Yes | Yes | Yes | 66.7% |
| Wudu_2013 | No | Yes | No | Yes | Yes | Yes | No | Yes | Yes | 66.7% |
| Rea_2016 | Yes | Yes | Yes | Yes | Yes | Yes | Yes | Yes | Yes | 100% |
